# A Novel Ultrasonic Leak Detection System in Nuclear Power Plants Using Rigid Guide Tubes with FCOG and SNR

**DOI:** 10.3390/s24206524

**Published:** 2024-10-10

**Authors:** You-Rak Choi, Doyeob Yeo, Jae-Cheol Lee, Jai-Wan Cho, Sangook Moon

**Affiliations:** 1 Nuclear System Integrity Sensing and Diagnosis Division, Korea Atomic Energy Research Institute (KAERI), 989-111 Daedeok-daero, Yuseong, Daejeon 34057, Republic of Korea; yrchoi@kaeri.re.kr (Y.-R.C.); yeody@kaeri.re.kr (D.Y.); jclee2@kaeri.re.kr (J.-C.L.); jwcho@kaeri.re.kr (J.-W.C.); 2 Department of Electrical and Electronic Engineering, Mokwon University, 88 Doanbuk-ro, Seo-gu, Daejeon 35349, Republic of Korea

**Keywords:** leak detection, nuclear reactor coolant system, rigid guide tube, FCOG, SNR

## Abstract

Leak detection in nuclear reactor coolant systems is crucial for maintaining the safety and operational integrity of nuclear power plants. Traditional leak detection methods, such as acoustic emission sensors and spectroscopy, face challenges in sensitivity, response time, and accurate leak localization, particularly in complex piping systems. In this study, we propose a novel leak detection approach that incorporates a rigid guide tube into the insulation layer surrounding reactor coolant pipes and combines this with an advanced detection criterion based on Frequency Center of Gravity shifts and Signal-to-Noise Ratio analysis. This dual-method strategy significantly improves the sensitivity and accuracy of leak detection by providing a stable transmission path for ultrasonic signals and enabling robust signal analysis. The rigid guide tube-based system, along with the integrated criteria, addresses several limitations of existing technologies, including the detection of minor leaks and the complexity of installation and maintenance. By enhancing the early detection of leaks and enabling precise localization, this approach contributes to increased reactor safety, reduced downtime, and lower operational costs. Experimental evaluations demonstrate the system’s effectiveness, focusing on its potential as a valuable addition to the current array of nuclear power plant maintenance technologies. Future research will focus on optimizing key parameters, such as the threshold frequency shift (Δf) and the number of randomly selected frequencies (N), using machine learning techniques to further enhance the system’s accuracy and reliability in various reactor environments.

## 1. Introduction

Leak detection in nuclear reactor coolant systems is a critical aspect of maintaining the safety and operational integrity of nuclear power plants. Effective leak detection systems help prevent potential catastrophic failures, reduce maintenance costs, and ensure the longevity of reactor components. Traditional methods of leak detection, such as acoustic emission sensors and spectroscopy, have been widely used. However, these methods often face challenges related to sensitivity, response time, and the ability to accurately locate leaks in complex piping systems [1,2]. The Leak-Before-Break (LBB) concept is an important methodology that complements these traditional leak detection technologies. LBB is designed to detect leaks in major piping systems of nuclear power plants before a fracture occurs. By enabling the detection and repair of cracks before they lead to catastrophic failure, LBB significantly enhances safety. This methodology allows nuclear reactors to prevent serious accidents, minimize downtime, and reduce maintenance costs [3,4]. In this study, we propose a novel approach to enhancing leak detection in nuclear reactor coolant pipes by incorporating a rigid guide tube. The guide tube is designed to be inserted into the insulation layer surrounding the coolant pipes, providing a direct pathway for ultrasonic signals to travel. This method aims to improve the sensitivity and accuracy of leak detection by reducing signal attenuation and noise, thereby facilitating early detection of leaks. The proposed rigid guide tube method addresses several limitations of current leak detection technologies. By providing a stable and direct transmission path for ultrasonic signals, the guide tube enhances the detection of minor leaks that might otherwise go unnoticed. Additionally, this approach simplifies the installation and maintenance processes, as the guide tube can be integrated into existing insulation layers without significant modifications to the reactor’s infrastructure [5]. Moreover, integrating the guide tube with advanced ultrasonic transducers and signal processing algorithms promises to deliver a highly reliable leak detection system. This system not only ensures early detection of leaks but also provides precise localization, enabling timely and targeted maintenance interventions [6,7]. The potential benefits of this method include increased reactor safety, reduced downtime, and lower operational costs, making it a valuable addition to the arsenal of technologies available for nuclear power plant maintenance [8].

In summary, this paper presents the design, implementation, and evaluation of a rigid guide tube-based leak detection system for nuclear reactor coolant pipes. The following sections detail the materials and methods used in the study, present the experimental results, and discuss the implications and potential applications of the proposed system. By advancing the capabilities of leak detection in nuclear reactors, this research contributes to the ongoing efforts to enhance the safety and efficiency of nuclear energy production.

## 2. Background and Related Work

### 2.1. Leak-Before-Break

The Leak-Before-Break concept is a crucial safety methodology in nuclear power plants designed to prevent catastrophic failures by ensuring that any potential cracks in the reactor coolant system’s piping lead to detectable leaks before a complete break occurs. This approach allows for timely maintenance and repair, thereby preventing severe accidents and minimizing downtime. The LBB concept originated in the 1960s, with initial applications in missile casing and pressure vessels using linear elastic fracture mechanics. Early foundational work by Irwin in 1961, followed by enhancements from Kobayashi in 1965, demonstrated that, under certain conditions, cracks can grow stably and produce detectable leaks before leading to catastrophic failure [9,10].

LBB methodologies have since been extensively applied in the nuclear industry, particularly within primary coolant systems. By employing LBB principles, nuclear reactors can detect leaks at an early stage, allowing operators to address issues before they escalate. This is facilitated through advanced monitoring techniques such as acoustic emission sensors and ultrasonic testing, which provide real-time data on the integrity of the piping systems [11,12]. The technical approach to LBB involves both load-controlled and displacement-controlled analyses. Load-controlled LBB deals with stresses from pressure or dead weight loads, typical in gas pressure lines. Displacement-controlled LBB is divided into local and global controls, where local control affects crack opening displacement minimally, while global control can induce ductile fracture even in rigid materials.

Regulatory bodies like the US Nuclear Regulatory Commission (NRC) have established guidelines for LBB implementation, including fracture mechanics analysis procedures and criteria to ensure cracks result in leaks before catastrophic failure. The NRC’s Standard Review Plan 3.6.3, developed in the 1980s, provides a comprehensive framework for LBB application in nuclear plants [13]. The benefits of LBB implementation in nuclear reactors include enhanced safety through early leak detection, reduced downtime due to proactive maintenance, and cost savings from preventing severe accidents and enabling timely repairs.

### 2.2. Acousto-Optic Leakage Monitoring

The acousto-optic leakage monitoring system for nuclear power plant main steam pipelines combines acoustic emission sensors and spectroscopy to detect leaks [5]. This system is designed to provide early warnings for leaks and improve the reliability of detection through method diversity and redundancy. The system includes acoustic emission sensors, micro-optical fiber probes, signal amplifiers, data acquisition units, laser transmitters, and spectrum analyzers, all connected through a network switch to a control unit and display unit.

Despite its advantages, there are several potential drawbacks to this method. The initial cost of installing such a complex system can be high due to the specialized equipment and extensive setup required. Installation is further complicated by the need to ensure the system operates effectively in high-temperature and high-radiation environments typical of nuclear power plants. Maintenance and repair also demand high technical expertise, increasing operational costs and dependency on specialized personnel. The system’s reliability is another concern, as it relies on the continuous and accurate functioning of numerous sensors and data processing units. Any failure in these components could lead to undetected leaks or false alarms, compromising plant safety. Additionally, the large volume of data generated by the sensors requires efficient processing and analysis to provide timely and accurate leak detection. Finally, the system must withstand extreme environmental conditions, which can challenge the durability and longevity of its components.

### 2.3. β-Ray Detection

The β-ray detection method for detecting small leaks in the reactor coolant system utilizes a silicon detector to directly detect beta particles emitted by specific radionuclides, such as 16N, present in the coolant. This method aims to address the limitations of existing leak detection systems that struggle to detect small leaks due to their insensitivity to slight changes in humidity, temperature, and radioactivity. In this method, the silicon detector offers high energy resolution for beta rays and a low gamma-ray response, making it suitable for operation within the reactor containment building. Experiments and simulations have shown that the system can achieve a detection threshold below 0.5 gallons per minute (gpm) [8]. The detection performance is evaluated by measuring the beta-ray count rate and comparing it to the background gamma-ray count rate. The silicon detector’s response is calibrated using beta sources like 90Sr/90Y, and simulations are conducted using the Monte Carlo N-particle extended code to estimate the beta-ray count rate for different leakage scenarios [14]. The system is designed to quickly transport leaked coolant to the detection chamber using an air pump, minimizing the delay between the occurrence of a leak and its detection.

However, there are several potential drawbacks to this method. The initial setup and calibration of the silicon detectors require specialized equipment and expertise. The system’s effectiveness is highly dependent on the accurate calibration and proper functioning of the detectors, which can be affected by the harsh conditions inside the reactor containment. Additionally, while the silicon detector is less sensitive to gamma rays, the presence of high-intensity gamma radiation can still interfere with the detection process, necessitating thorough shielding and background correction. Overall, the β-ray detection method represents a sophisticated approach to detecting small leaks in the reactor coolant system, offering significant improvements in sensitivity and response time over traditional methods. However, it also presents challenges related to setup complexity, maintenance, and operational reliability in the demanding environment of a nuclear reactor.

### 2.4. Probabilistic Evaluations

The probabilistic evaluation method using the Extremely Low Probability of Rupture (xLPR) code is applied to Pressurized-Water Reactor (PWR) piping systems to ensure that they maintain an extremely low probability of rupture, even in the presence of primary water stress corrosion cracking [15,16]. This approach involves using the xLPR code to analyze individual welds within these systems, aggregating the results to a system-level analysis. This meets the four requirements of the General Design Criteria (GDC), which obligate that safety systems withstand environmental and dynamic effects such as temperature fluctuations, pressure changes, seismic events, and pipe ruptures [17]. The evaluation includes variables such as normal operating loads, pressures, and temperatures, selecting the most conservative inputs to encompass all in-service dissimilar metal welds. The analysis measures several quantities of interest, including the probabilities of first crack, first leak, rupture with and without in-service inspection, and the ratio between critical crack length at rupture and detectable leakage crack length. The study found that even under conservative assumptions, the probability of rupture for the evaluated PWR piping systems remains extremely low, supporting the continued use of the LBB approach.

However, the probabilistic evaluation method presents several challenges. The initial setup involves complex simulations and requires detailed knowledge of the material properties and operational conditions. The analysis also heavily depends on the accuracy of input data and assumptions, such as weld residual stress profiles and initial flaw sizes, which can significantly impact the results. Additionally, the method requires sophisticated software and computational resources, along with expertise in probabilistic fracture mechanics, to perform and interpret the evaluations accurately. While the probabilistic evaluation method provides a robust framework for assessing the integrity of PWR piping systems under various scenarios, it also demands significant resources and expertise to implement effectively.

## 3. Enhanced Acoustic Leak Detection System

### 3.1. Current Acoustic Leak Detection Technology

Leak detection technology for reactor coolant piping located within the Reactor Coolant Pressure Boundary (RCPB) of nuclear power plants is crucial under the LBB detection concept. To detect leaks in reactor coolant piping, ultrasonic acoustic leak detection technology is utilized. When a leak occurs at a defect site, such as a crack or perforation in the piping, ultrasonic waves are generated. These waves are collected by acoustic sensors, and the signals are processed to detect the leak. Currently, an Acoustic Leak-Monitoring System (ALMS) is operated to detect leaks in the reactor coolant piping of nuclear power plants. Acoustic sensors are attached to the surface of the reactor coolant piping, and the fixtures for attaching the sensors are welded in place. The acoustic sensors are connected to signal processing devices using hardline cables, which are highly resistant to high temperatures and radiation, and softline cables [18]. These cables can extend for dozens of meters. Due to the costs associated with welding the fixtures for sensor attachment and the installation of signal transmission cables, it is impractical to install many sensors. In practice, nuclear power plants apply methods that measure changes in equipment vibrations and humidity variations during a leakage event. Vibration measurements primarily use Acoustic Emission (AE) sensors; however, due to their high frequency processing band, these sensors present challenges in wireless implementation due to power consumption issues, and their installation on equipment surfaces is constrained by cabling and sensor placement. In the case of humidity measurement, the accuracy is compromised, as changes in humidity are measured far from the leakage point due to the generation of high-pressure steam [19].

The reactor coolant piping is covered with insulation to prevent heat loss. Therefore, detecting leaks in the reactor coolant piping from outside the insulation without directly attaching sensors to the piping surface is practically impossible. Consequently, the current acoustic leak detection system faces limitations regarding the number of sensors that can be installed and the associated costs, as well as challenges in detecting leaks through the insulation layer.

### 3.2. Acoustic Signal Forwarding with Rigid Guided Tubes

The core concept we propose is the insertion of rigid guide tubes into the insulation surrounding the reactor coolant piping, allowing for the placement of probes to measure leakage sounds at various points. Our focus is on pressurized water reactors (PWRs), which convert H_2_O into dry steam to drive turbines. In these systems, water leaking under pressures between 75 and 150 bar and temperatures of 150–300 °C rapidly vaporizes, effectively mimicking a gas leak (air). For the purpose of this study, we treat the coolant as analogous to air. In the event of a coolant leak caused by a defect in the reactor coolant piping, the ultrasonic leakage sound propagates through the gap between the insulation and the reactor coolant piping surface. This ultrasonic sound is transmitted outside the insulation via the rigid guide tubes. By attaching ultrasonic leakage detectors to the ends of the guide tubes positioned outside the insulation, leaks in the reactor coolant piping can be detected.

**Figure 1:**
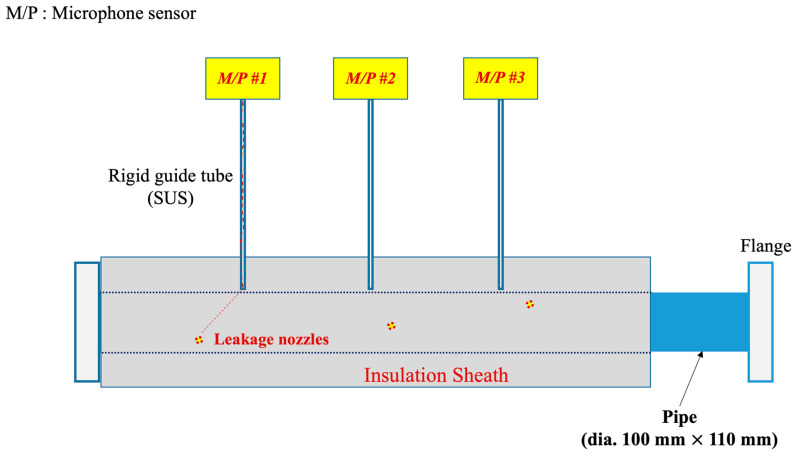
Concept of leak detection with rigid guide tubes for piping covered with insulation.

Figure 1 illustrates the leak detection concept proposed in this study for piping covered with insulation, as well as the experimental design diagram. M/P denotes acoustic (microphone) sensors, and the figure shows a total of three M/P sensors connected to the external ends of the rigid guide tubes. Three leakage nozzles (LN #1, #2, and #3) are designated on the pipe, each positioned sequentially near one of the three installed rigid guide tubes. When a leak occurs at a pipe defect, the ultrasonic leakage sound propagates through the gap between the pipe surface and the insulation, travels through the interior of the inserted rigid guide tubes, and is received by the M/P sensors attached to the opposite ends. The sensors then process the signal to detect the leak.

**Figure 2:**
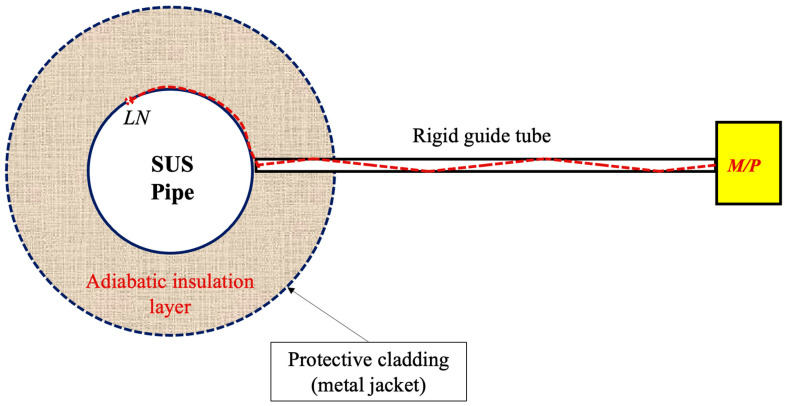
Cross-sectional view of the insulation-sheathed pipe connected to an M/P sensor via a rigid guide tube.

Figure 2 is a cross-sectional view of Figure 1. LN refers to the leakage nozzle, illustrating a single leak point connected to an M/P sensor via a rigid guide tube. The red dashed lines in the figure show the paths of the ultrasonic waves generated at the leakage nozzle as they propagate through the gap between the pipe and the insulation and are transmitted to the M/P sensor outside the insulation through the rigid guide tube.

As depicted subtly in the figure, the edge of the rigid guide tube is designed not to make direct contact with the pipe surface. Consequently, vibrations from the pipe are not transmitted to the M/P sensor through the rigid guide tube, allowing only the ultrasonic signals generated by the leak to be detected. Since the end of the rigid guide tube is spaced away from the pipe surface, process noise such as fluid-induced vibrations and cavitation occurring during normal operation of the nuclear plant does not reach the M/P sensor. This design feature is expected to result in high leak detection sensitivity.

### 3.3. Enhanced FCOG for Leak Detection

The Frequency Center of Gravity (FCOG) is a concept that represents the mean frequency of a frequency spectrum. It indicates the centroid of energy distribution within the frequency spectrum of a signal and is widely used in signal processing and analysis. FCOG is particularly useful in applications such as leak detection, where understanding the distribution of frequency components in a signal is crucial [20,21]. FCOG is defined as the weighted average of the frequency components, where each frequency component *f_i_* is weighted by its corresponding energy or spectral density *A*(*f_i_*). Mathematically, it is expressed as in Equation (1):

(1) $$FCOG=\frac{\sum_{i}^{n}f_{i}\times A(f_{i})}{\sum_{i}^{n}A(f_{i})},\;$$

where:

- *f_i_* represents the frequency component *I*;
- *A*(*f_i_*) denotes the energy or spectral density at frequency component *f*_i_;
- *N* is the total number of frequency components.

FCOG represents the frequency range where the signal’s energy is concentrated within the spectrum. Similar to the concept of the center of mass in physical objects, where the center of mass is the point at which the entire mass of an object is balanced, FCOG is the point in the frequency domain where the energy distribution of the signal is balanced. FCOG simplifies signal analysis by providing a single value that summarizes the frequency characteristics of the signal. This is particularly useful for analyzing complex signals with multiple frequency components, making it easier to understand the signal’s overall frequency distribution and detect any shifts or changes in its characteristics.

To detect pipe leaks by sensing ultrasonic waves, we apply the concept of FCOG and an additional method described below:

The M/P sensor will randomly select *N* frequencies from the entire frequency range within its response characteristics. If the Signal-to-Noise Ratio (S/N) for at least *N*/2 of these selected frequencies is greater than 2, a leak is determined to have occurred. This method leverages the characteristic of the ultrasonic waves excited by a leak, which exhibit properties of white noise, thereby presenting energy components across almost all frequencies.

The characteristic of white noise is that it has equal energy across all frequency bands. Therefore, the frequency spectrum of a signal containing white noise is uniformly and evenly distributed. FCOG is a reliable metric for summarizing the frequency characteristics of a signal. When a leak occurs, the shift in the frequency spectrum will cause the FCOG to move. By monitoring changes in the FCOG, significant deviations from the background noise can reliably indicate the presence of a leak. Randomly selecting frequencies allows for a more comprehensive sampling of the entire frequency spectrum, avoiding bias towards any specific frequency band. This ensures that the analysis accurately reflects the overall frequency characteristics of the signal. Here, the S/N ratio is a critical measure of signal quality. When a leak occurs, the energy in certain frequency bands increases sharply, leading to higher S/N ratios. If more than half of the selected frequencies exhibit S/N ratios greater than the threshold, it strongly indicates the presence of a leak.

Due to the uniform energy distribution of white noise, randomly selected frequencies are likely to capture the leak signal. This reduces dependency on specific frequency bands, enhancing the reliability of leak detection. Analyzing multiple frequencies reduces the likelihood of errors that might occur when relying on a single frequency. Observing consistent patterns (such as increased S/N ratios) across multiple frequencies provides strong evidence of a leak.

By combining the FCOG method with random frequency selection and S/N ratio analysis, we can develop a robust and theoretically sound approach to detecting leaks in insulated piping systems. This dual-method strategy capitalizes on the strengths of both techniques, ensuring high reliability and accuracy in leak detection.

### 3.4. Leak Detection Criteria

We propose a novel, combined approach for detecting leaks in piping covered with insulation. This approach integrates the established method of FCOG shift detection with a new S/N-based criterion. A leak is confirmed if either of the following criteria is satisfied, enhancing the reliability and accuracy of the detection process.

Criterion 1: FCOG Shift

The FCOG is calculated, and if a shift in the FCOG is detected, it is determined that a leakage event has occurred. Since the ultrasonic waves excited by a leak exhibit characteristics of white noise, energy components appear across almost all frequencies. When a leak occurs, there is a high probability that the FCOG will shift. Therefore, by calculating the FCOG and identifying significant deviations from the background noise FCOG, a leak is considered to be detected.

$$Leak_{EVENT}=True,\;if\;\left|FCOG_{Leak}-FCOG_{B/G}\right|>\Delta f,$$

where:

- FCOG_Leak_ represents the FCOG for the detected leak signal;
- FCOG_B/G_ represents the FCOG for the background noise signal;
- ∆*f* is the threshold frequency that defines the minimum deviation required to determine a leak event.

Criterion 2: S/N Ratio in Random Frequencies

The M/P sensor will randomly select N frequencies from its entire response range. If the S/N ratio of at least N/2 of these selected frequencies exceeds 2, a leak is determined to have occurred. This method is effective because the ultrasonic waves generated by a leak exhibit the characteristics of white noise, distributing energy across almost all frequency bands. Since white noise distributes energy uniformly across all frequency bands, analyzing only a subset of frequencies can still accurately reflect the overall signal characteristics. Evaluating the S/N ratio of the selected frequencies is sufficient to assess the general state of the signal.

Reasons for not evaluating the S/N ratio at all measured frequencies are as follows. First, assessing the S/N ratio at all frequencies can lead to false detections due to transient noise or external interference, as noise might temporarily increase at certain frequencies, distorting the overall SNR assessment. Second, calculating the S/N ratio at all frequencies is computationally expensive and inefficient for real-time processing. Therefore, sampling key frequencies allows for efficient use of computational resources while still capturing essential signal characteristics. Third, due to the properties of white noise, which distributes energy uniformly across all frequency bands, analyzing a subset of frequencies can adequately represent the overall signal characteristics. Lastly, evaluating the S/N ratio at the sampled frequencies reduces the likelihood of false positives and false negatives. If more than half of the selected frequencies exceed the S/N threshold, it indicates a high probability of an actual leak.

The reason for using N/2 as the threshold is to ensure statistical reliability, better reflect signal characteristics, provide experimental validity, and reduce the probability of errors. Requiring that more than half of the selected frequencies have an S/N ratio exceeding 2 ensures statistically reliable results. This criterion minimizes random variations in frequency sampling and reduces the chances of false detections. White noise spreads energy uniformly across all frequencies, so a leak signal is likely to be detected across many frequencies. Ensuring that over half the frequencies have a high S/N ratio indicates the pervasive presence of the leak signal. Various experiments have demonstrated that the N/2 criterion provides higher reliability [22]. If more than half of the sampled frequencies exceed the S/N threshold, it strongly suggests an actual leak. The N/2 criterion offers more consistent results, better controlling experimental conditions and ensuring reliable data collection. Lastly, requiring that more than half of the frequencies exceed the S/N threshold reduces the likelihood of errors. This approach excludes temporary noise variations and more accurately reflects the actual signal. Considering the above grounds, we propose Criterion 2 as follows:

$$Leak_{EVENT}=True,\;if\;\frac{1}{N}\sum_{i=1}^{N}S/N\left(f_{i}\right)>2\;and\;\frac{Count\left(f_{i}|S/N\left(f_{i}\right)>2\right)}{N}\;\geq \;\frac{1}{2},$$

where:

- $S/N\left(f_{i}\right)\;$ represents the Signal-to-Noise Ratio at frequency f*_i_*;
- N is the total number of randomly selected frequencies;
- $Count\left(f_{i}|S/N\left(f_{i}\right)>2\right)$ denotes the number of frequencies for which the S/N ratio exceeds 2.

By combining the FCOG method with random frequency selection and S/N ratio analysis, we propose a robust and theoretically sound approach to detecting leaks in insulated piping systems. This dual-method strategy capitalizes on the strengths of both techniques, ensuring high reliability and accuracy in leak detection.

## 4. Experiments and Results Analysis

### 4.1. Experimental Setup

**Figure 3:**
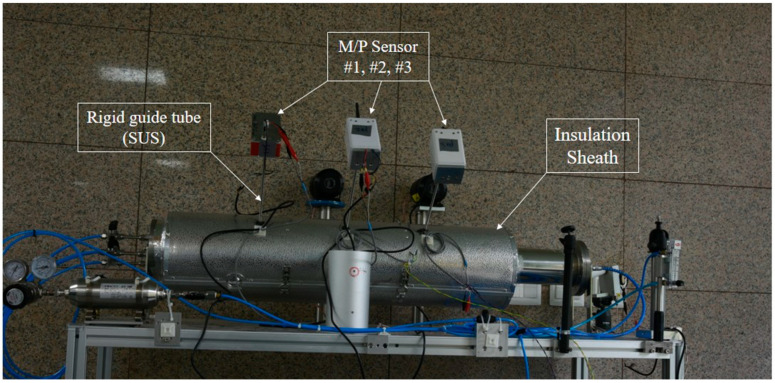
Experimental setup of leak detection with rigid guide tubes for piping covered with insulation.

Figure 3 illustrates the experimental setup designed to test the concept depicted in Figure 1. Both the rigid guide tube and the pipe are made of SUS (Stainless Steel Use), with the most common types being SUS304, SUS316 (low carbon version), and SUS430 (ferritic stainless steel containing only chromium). For this experiment, SUS304 was chosen as the material for the pipe mock-up due to its general-purpose nature, affordability, and ease of fabrication [23]. The rigid guide tube also utilizes commercially available products, specifically using the most common 1/4”-diameter SUS tubing. Given that the wall thickness of a 1/4” pipe is 1 mm, the internal diameter becomes approximately 4.35 mm after subtracting 2 mm from the external diameter of 6.35 mm (1/4”). We assumed in this experiment that the ultrasonic waves generated by the leak would propagate through the 4.35 mm internal diameter space, and the test apparatus was configured accordingly.

**Figure 4:**
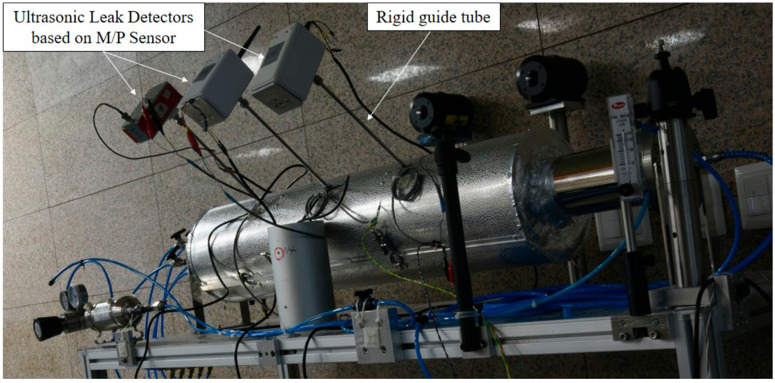
Side view of the experimental setup shown in Figure 3, illustrating the arrangement of the rigid guide tube, M/P sensors, and insulation sheath.

To complement this, a side view of the experimental setup is provided in Figure 4. This side perspective offers a clearer view of the arrangement and positioning of key components such as the rigid guide tube, M/P sensors (KAERI, Daejeon, Republic of Korea), and insulation sheath. Particularly, the side view illustrates the spatial relationships between these elements and demonstrates how the ultrasonic leak detectors (ULDs #1, #2, and #3 (KAERI, Daejeon, Republic of Korea)) are positioned relative to the piping and insulation. This perspective shows the overall configuration and the functionality of the experimental setup, emphasizing how the components work together to detect leaks.

The experimental setup in Figure 3 and Figure 4 was constructed according to the design specifications outlined in Figure 1 and Figure 2. Specifically, the edge of the rigid guide tube was designed not to make direct contact with the pipe surface. This ensures that vibrations from the pipe are not transmitted through the rigid guide tube to the M/P sensors, allowing only the ultrasonic signals generated by leaks to be detected. This implementation prevents process noise, such as fluid-induced vibrations and cavitation occurring during normal operation of the nuclear plant, from reaching the M/P sensors, thereby enhancing leak detection sensitivity.

### 4.2. Leak Point near ULD #2 (LN #2, 0.5 mm Leakage Nozzle, 200 kPa)

This experimental evaluation focuses on detecting leaks near the center point in the piping system shown in Figure 1 under controlled conditions, specifically at a gauge pressure of 200 kPa with a 0.5 mm leakage at LN #2. This setup aimed to simulate a realistic leak scenario in nuclear reactor coolant systems, providing a practical assessment of the proposed leak detection methodology using rigid guide tubes. This leak detection experiment was conducted with the LN #2 leak point within the test pipe shown in Figure 1. The setup included a rigid guide tube inserted into the insulation layer, directly facilitating the transmission of ultrasonic signals from the leak source (LN #2) to the external sensor ULD #2. The acoustic sensor was strategically placed at the end of the guide tube, allowing for efficient capture of the emitted ultrasonic wave.

**Figure 5:**
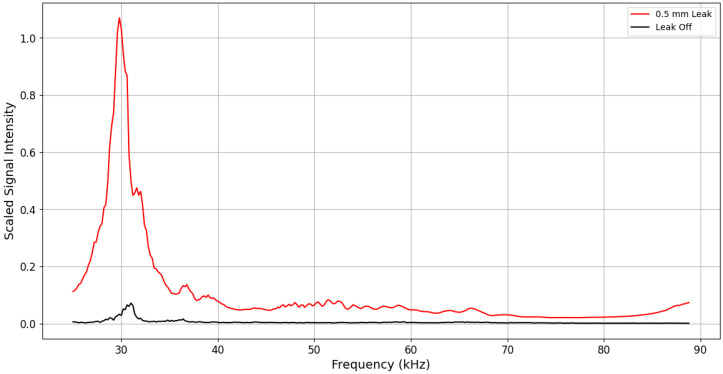
Power spectrum comparison between 0.5 mm leak at LN #2 and leak-off measured by ULD #2 at a gauge pressure of 200 kPa.

Figure 5 represents the frequency spectrum analysis with and without a leak event. The M/P sensor measures sound pressure level (SPL), and the signal output unit is V/SPL (depending on the sensitivity, microvolt/SPL or nanovolt/SPL). The signal is processed and converted into the power spectrum via FFT (Fast Fourier Transform) in frequency units, which is shown on the vertical axis. Strictly speaking, since it is power, the unit should be watts; however, to focus on understanding the differences rather than converting to exact power, it is broadly referred to as scaled intensity, which signifies energy intensity.

The ultrasonic signals were analyzed across various frequency ranges, focusing particularly on those above 25 kHz. This threshold was chosen based on the understanding that ultrasonic frequencies above 25 kHz are more likely to propagate effectively through the insulation and guide tube, reaching the sensors with minimal attenuation. The analysis was divided into three specific frequency bands:

Lower Frequency Band (25–46.6 kHz):

In this range, the signals exhibited a significant increase in amplitude in the presence of a leak. The Signal-to-Noise Ratio (SNR) for this band was calculated to be approximately 25.95, indicating a clear and detectable difference between leak and no-leak conditions. This high SNR suggests that this frequency range is highly sensitive to leaks, making it a critical component for early detection.

Middle Frequency Band (46.8–67.4 kHz):

The middle frequency band also showed a notable difference between the leak and no-leak scenarios, with an SNR of 23.33. While slightly lower than the lower frequency band, this range still provides a reliable signal for leak detection. The consistency in signal differences across this band underscores its usefulness in the leak detection process, ensuring that even minor leaks are effectively captured.

Upper Frequency Band (67.6–88.8 kHz):

In the upper frequency range, the SNR was observed to be 23.53. This band demonstrated a consistent, though slightly reduced, amplitude difference compared to the lower bands. Despite this reduction, the high SNR in this band indicates that it remains a viable range for detecting leaks. The data suggest that, while the attenuation might be higher at these frequencies, the detection system is still capable of distinguishing between leak and non-leak conditions effectively.

#### 4.2.1. FCOG Shift Analysis (Leakage @ LN #2)

**Figure 6:**
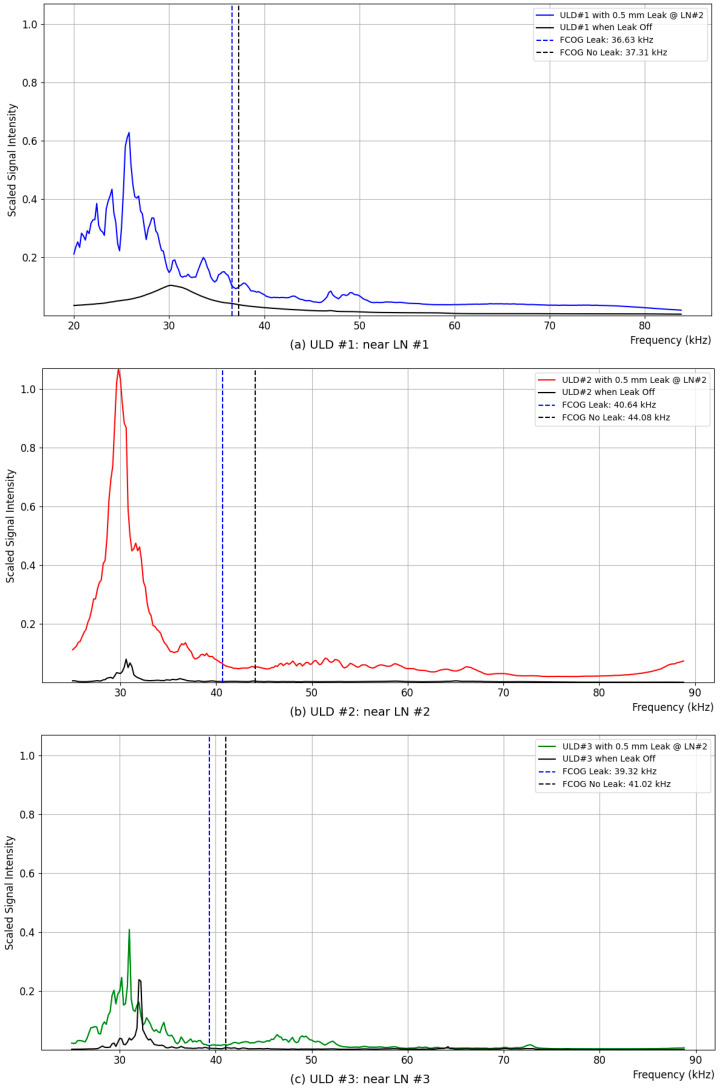
FCOG measured at three different ULD points with a 0.5 mm leak at LN #2 and leak-off at a gauge pressure of 200 kPa; (**a**) ΔFCOG = 0.68 kHz @ ULD #1; (**b**) ΔFCOG = 3.44 kHz @ ULD #2; (**c**) ΔFCOG = 1.7 kHz @ ULD #3.

Figure 6 compares the ultrasonic sensing data measured by ULD #1, #2, and #3 when there is a leakage and when there is no leakage at the location of leakage nozzle #2, while the SUS pipe indicated in Figure 1 is passing air at a gauge pressure of 200 kPa. In the case of leakage, the air is discharged through a 0.5 mm nozzle, taking approximately 280 s, and the leakage signal values are calculated as the average over 280 s for each frequency band. When there is no leakage, the background noise is measured, assuming that the compressed air stored in the pipe has completely escaped after 1000 s, and the average values measured over approximately 1700 s thereafter, deemed a sufficient time, are calculated and displayed.

(a) shows the ultrasonic intensity measured by ULD #1 sensor, located to the left of the leakage position, when there is and is not a leak. If the audible range signals are processed together, the signal strength in the audible range is so large that the high-frequency signals are overshadowed. To compensate for this, the audible range was filtered, and only the ultrasonic range above 20 kHz was processed. The ULD #1 sensor measured values in the range from 20 kHz to 83.8 kHz. The FCOG was calculated according to Equation (1). The FCOG was 36.3 kHz when there was a leak and 37.31 kHz when there was no leak, with a difference of 0.68 kHz. (b) shows the ultrasonic intensity measured by ULD #2 sensor, located closest to the leakage position, when there is and is not a leak. The ultrasonic sensor of ULD #2 experimentally varied the frequency band, excluding the audible range from the signal processing stage up to 25 kHz and measuring values in the range from 25 kHz to 88.8 kHz. The FCOG was 40.64 kHz when there was a leak and 44.08 kHz when there was no leak, with the highest difference calculated at 3.44 kHz. (c) shows the ultrasonic intensity measured by ULD #3 sensor, located to the right of the leakage position, when there is and is not a leak. The measurement range of ULD #3 was the same as that of ULD #2, measuring values from 25 kHz to 88.8 kHz. The FCOG was 39.32 kHz when there was a leak and 44.08 kHz when there was no leak, with a difference of 1.7 kHz.

Although the absolute values of the FCOG shifts measured at the three ULD locations differ, they share the common characteristic of a shift in the frequency center of gravity. The determination of a leakage event can be made by setting the value of Δf according to the characteristics of the pipe, as suggested in the FCOG Shift criterion 1.

#### 4.2.2. S/N Ratio Analysis (Leakage @ LN #2)

**Figure 7:**
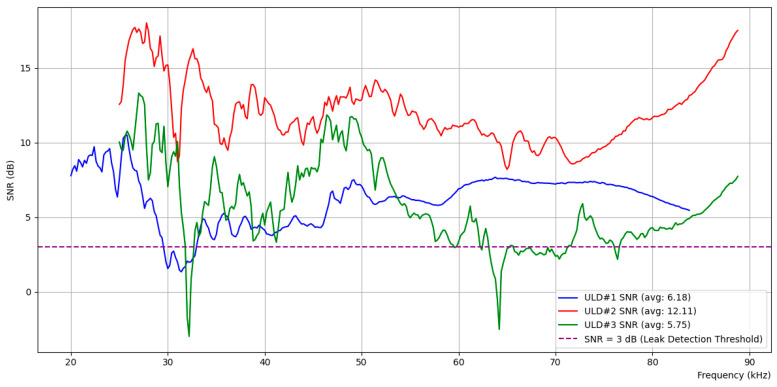
SNR analysis across ULD sensors for 0.5 mm leak at LN #2.

Figure 7 shows the SNR calculated from the average ultrasonic measurements across different frequency bands over time for ULDs #1, #2, and #3, both with and without a 0.5 mm leak at LN #2. The SNR is expressed in decibels (dB) according to Equation (2) below:

(2) $${SNR}_{dB}=10\log_{10}\left({SNR}_{ratio}\right)$$

The SNR calculated at ULD #1 (blue) shows an average value of 6.18 dB, while ULD #2 (red), which is closest to LN #2, shows the highest average value of 12.11 dB. ULD #3 (green), positioned on the right side, recorded an average value of 5.75 dB. According to the proposal in Criterion 2, we set the threshold S/N ratio to 2, which, when converted to decibels, corresponds to 3 dB, as indicated by the purple dashed line in the figure. The SNR measured at ULD #2 exceeds 3 dB across the entire frequency spectrum, while ULD #1 and ULD #3 also exceed the 3 dB threshold in approximately 80% to 90% of the spectrum, as can be observed visually. This implies that, when checking the SNR for randomly selected N frequencies, more than N/2 frequencies exceed the threshold SNR of 2 (3 dB) at all ULDs, indicating a leakage. The results suggest that the leakage point is particularly close to ULD #2, where the probability of exceeding the threshold is 100%.

### 4.3. Leak Point near ULD #3 (LN #3, 1.0 mm Leakage Nozzle, 200 kPa)

In this experiment, a 1.0 mm leak at the right-side leak point (LN #3) in the piping system shown in Figure 1 was conducted under a gauge pressure of 200 kPa. The experimental setup was similar to that of the experiment in Section 4.1, except that the air was discharged through a 1.0 mm nozzle, with the leakage process taking approximately 220 s. During this period, the ultrasonic signals were captured and averaged over this time span for each frequency band. When no leakage occurred, background noise was measured after ensuring that the compressed air had completely escaped from the pipe, and the average values were calculated over a subsequent 180 s, which was deemed sufficient for the background noise measurement. The frequency spectrum was analyzed by comparing the conditions with and without the leak event.

#### 4.3.1. FCOG Shift Analysis (Leakage @ LN #3)

**Figure 8:**
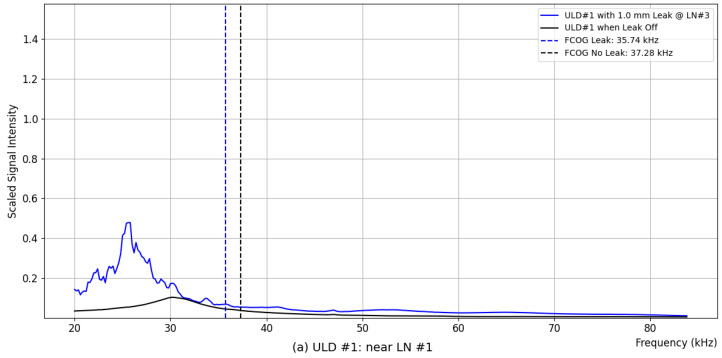

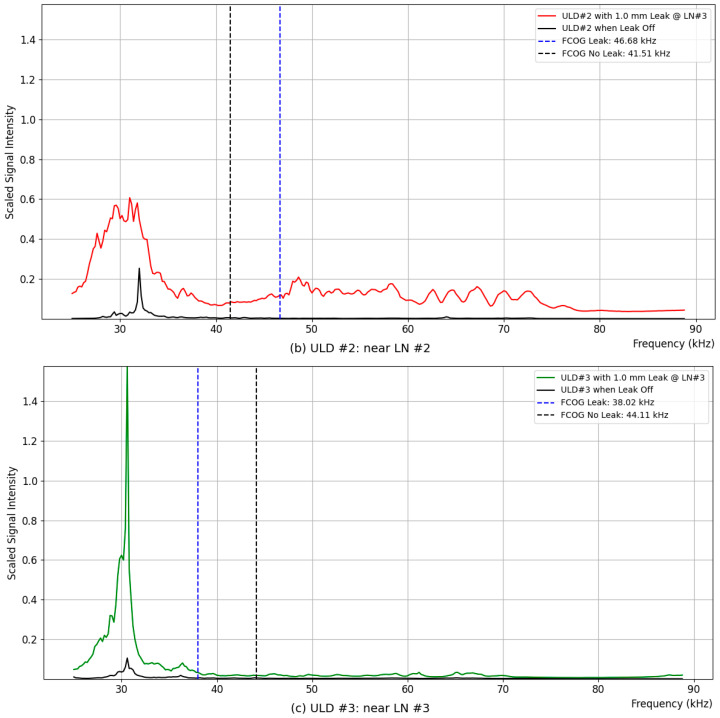
FCOG measured at three different ULD points with a 1.0 mm leak at LN #3 and leak-off at a gauge pressure of 200 kPa; (**a**) ΔFCOG = 1.54 kHz @ ULD #1; (**b**) ΔFCOG = 5.17 kHz @ ULD #2; (**c**) ΔFCOG = 6.09 kHz @ ULD #3.

Figure 8 compares the ultrasonic sensing data measured by ULDs #1, #2, and #3 when there is a leakage and when there is no leakage at the location of LN #3. (a) shows the ultrasonic intensity captured by ULD #1, positioned to the left of the leakage point, with and without the leakage. Similarly to the previous experiment, the audible range signals were filtered out to avoid overshadowing the ultrasonic frequencies. The ULD #1 sensor measured values in the range from 20 kHz to 83.8 kHz. The FCOG was calculated according to Equation (1). The FCOG during leakage was found to be 35.74 kHz, while it was 37.28 kHz when there was no leakage, resulting in a shift of 1.54 kHz. (b) presents the ultrasonic intensity captured by ULD #2, which is positioned between LN #1 and LN #3. The ultrasonic sensor of ULD #2 was set to measure frequencies from 25 kHz to 88.8 kHz, excluding the audible range during the signal processing stage. The FCOG during the leakage was 46.68 kHz, compared to 41.51 kHz in the absence of leakage, resulting in a shift of 5.17 kHz. (c) displays the ultrasonic intensity recorded by ULD #3, located closest to the leakage point LN #3. The measurement range for ULD #3 was identical to that of ULD #2, covering the frequency range from 25 kHz to 88.8 kHz. The FCOG was 38.02 kHz during the leakage and 44.11 kHz without leakage, showing a shift of 6.09 kHz.

The results of this experiment demonstrate that, despite the difference in absolute FCOG shifts across the three sensors, there is a consistent shift in the frequency center of gravity when a leak is present. The observed FCOG shifts support the identification of leakage events as per Criterion 1: FCOG shift. By setting the value of Δf appropriately for the pipe characteristics, the detection of leakage events can be reliably determined based on the significant deviations observed in the FCOG during this experiment. The larger shifts in FCOG in this experiment compared to the previous one reflect the larger nozzle size of 1.0 mm, which contributed to the increased energy distribution across the frequency spectrum.

#### 4.3.2. S/N Ratio Analysis (Leakage @ LN #3)

**Figure 9:**
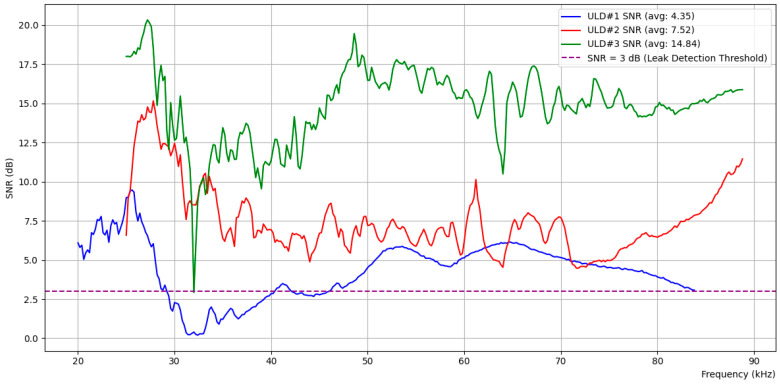
SNR analysis across ULD sensors for 1.0 mm leak at LN #3.

Figure 9 shows the SNR calculated from the average ultrasonic measurements across various frequency bands over time for ULDs #1, #2, and #3 in the presence of a 1.0 mm leak at LN #3. In this scenario, the SNR calculated at ULD #1 (blue) showed an average value of 4.35 dB, while ULD #3 (red), which is positioned closest to LN #3, displayed the highest average SNR of 14.84 dB. ULD #2 (green), located further from the leakage site but still within proximity, recorded an average SNR of 7.52 dB.

According to the proposal in Criterion 2, we set the threshold S/N ratio to 2, which corresponds to 3 dB when converted to decibels, as indicated by the purple dashed line in the figure. The SNR observed at ULD #3 exceeded 3 dB across the entire frequency spectrum except one point (probably a glitch), clearly indicating its proximity to the leakage point. Meanwhile, ULD #1 exceeded the 3 dB threshold in more than 80% of the spectrum, reflecting its position relative to the leak. ULD #2, despite having a lower average SNR, consistently maintained an SNR above the 3 dB threshold across the entire frequency range. These results suggest that, while all three sensors successfully detected the leakage, the highest average SNR observed at ULD #3 strongly indicates that the leak source was closest to this sensor. The consistency of SNR values above the threshold at ULD #2 further supported the detection of the leak, but the higher SNR at ULD #3 was critical in determining the leak’s precise location. This experimental observation aligns with Criterion 2, where the sensor with the highest SNR is likely closest to the leak, providing a reliable method for both detecting and localizing the leak.

### 4.4. Future Directions in Parameter Optimization Using Machine Learning

To determine the optimal values for ∆f, which represents the threshold frequency shift in Criterion 1, and N, the number of randomly selected frequencies in Criterion 2, machine learning techniques can be effectively utilized. These parameters are crucial for the accurate detection and localization of leaks using the FCOG shift and S/N ratio methods, respectively. By applying machine learning, it is possible to systematically explore large parameter spaces and identify the most effective settings for these criteria based on historical data or simulated leak scenarios.

The process begins with the collection of a comprehensive dataset that includes various leak and non-leak scenarios. This dataset encompasses measurements of FCOG shifts and S/N ratios across different frequencies and sensors, recorded under different conditions and for different leak sizes. Each data point is labeled to indicate whether a leak was present and, if so, the exact location of the leak. The collected data need to be preprocessed to ensure consistency, which involves cleaning the data, normalizing measurements, and removing any outliers that might skew the model’s learning process. Once a clean and well-prepared dataset is available, a supervised machine learning approach is applied. For determining the optimal value of ∆f, regression models such as Ridge regression, Lasso regression, or Elastic Net can be used [24]. These models are well-suited for capturing the relationships between the FCOG shifts and the leak detection criteria, allowing for the identification of the threshold that best distinguishes between leak and non-leak events.

For finding the optimal value of N, classification models such as logistic regression, decision trees, or random forests would be appropriate [25]. These models can be trained to predict the presence of a leak based on subsets of S/N ratios across randomly selected frequencies. By exploring different values of N, the model can determine the number of frequencies that maximizes the accuracy of leak detection while minimizing the likelihood of false positives. During model training, hyperparameter tuning techniques like grid search or Bayesian optimization can be employed to fine-tune the models [26]. This process would help in identifying the specific values of ∆f and N that optimize the model’s performance, ensuring that the leak detection system operates with high sensitivity and specificity. After training, the model’s performance must be validated using a separate validation dataset. This step is crucial to ensure that the model generalizes well to new, unseen data. Cross-validation techniques are used here to provide a robust assessment of the model’s reliability. Finally, the model is tested on a holdout test dataset to confirm its accuracy in real-world scenarios.

Once the optimal values for ∆f and N have been identified, the values are implemented within the leak detection system. However, it is important to recognize that environmental conditions and operational factors might change over time. Therefore, a continuous learning framework can be established, allowing the model to be periodically retrained with new data. This approach ensures that the system remains effective and adapts to any changes, maintaining its high accuracy and reliability in detecting leaks.

## 5. Conclusions

In this study, we proposed a novel approach for enhancing leak detection in nuclear reactor coolant pipes using a rigid guide tube-based system. This method aims to improve the sensitivity and accuracy of leak detection by providing a stable and direct transmission path for ultrasonic signals, thereby addressing several limitations of existing technologies. The proposed system has the potential to significantly enhance the safety of nuclear power plants by facilitating early leak detection, reducing downtime, and lowering operational costs.

The acousto-optic leakage monitoring system described in Section 2.2 combines acoustic emission sensors with spectroscopy to provide early leak detection. While this system offers the advantage of method diversity and redundancy, it is associated with high installation costs, complex setup requirements in high-temperature and high-radiation environments, and reliance on the continuous operation of multiple sensors and data-processing units. These factors can limit its reliability and increase maintenance complexity. The β-ray detection method outlined in Section 2.3 utilizes silicon detectors to detect beta particles emitted by specific radionuclides in the coolant. This method provides high sensitivity for detecting small leaks, but presents challenges such as the need for precise calibration, potential interference from high-intensity gamma radiation, and operational difficulties in the harsh reactor environment. The system’s effectiveness is highly dependent on the accurate calibration and proper functioning of the detectors, which can be compromised in such conditions. The probabilistic evaluation method discussed in Section 2.4 employs the xLPR code to ensure an extremely low probability of rupture in PWR piping systems. This approach provides a robust framework for assessing the integrity of reactor piping under various scenarios, but it requires complex simulations, detailed material and operational data, and sophisticated computational resources. Additionally, the accuracy of this method is highly contingent on the quality of input data and assumptions, which can significantly affect the results.

In contrast, the rigid guide tube-based leak detection system proposed in this study offers several distinct advantages. It is relatively simple to install and maintain, as it can be integrated into existing insulation layers with minimal modifications to the reactor infrastructure. The guide tube provides a stable and direct path for ultrasonic signals, allowing for the detection of minor leaks that may otherwise go unnoticed by traditional methods. Furthermore, the proposed leak detection criteria promise a highly reliable leak detection system that not only ensures early detection, but also allows for precise localization of leaks, enabling timely and targeted maintenance interventions.

In conclusion, the rigid guide tube-based leak detection system proposed in this research represents a significant advancement in the technology available for nuclear reactor maintenance. By overcoming the limitations of existing detection methods, this approach enhances the safety and efficiency of nuclear power plant operations. Future work will focus on the experimental validation of this system’s performance and its parameters across various reactor environments, with the goal of further optimizing its capabilities for widespread adoption in the nuclear industry.

## Data Availability

The datasets generated from the current study are available from the corresponding author upon reasonable request.

## References

[1] Ballesteros A., Sanda R., Peinador M., Zerger B., Negri P., Wenke R. (2014). Analysis of events related to cracks and leaks in the reactor coolant pressure boundary. Nucl. Eng. Des..

[2] El-Sayed M., El Domiaty A., Mourad A.-H.I., Thekkuden D.T. (2022). Leak before break fracture assessment of pressurized unplasticized PVC pipes with axial surface crack: Experimental and analytical analysis. Eng. Fract. Mech..

[3] Wilkowski G. (2000). Leak-Before-Break: What Does It Really Mean?. J. Press. Vessel Technol..

[4] Clarion Energy Content Directors (2010). An Historical Survey of Leak-Before-Break in Nuclear Plant Piping. Power Eng..

[5] Niu T., Jiang H., Yan J., Liu C., Shi W., Xia S., Cai Y., Zhan M., Li F., Zhang M. (2023). Acousto-Optic Leakage Monitoring System for Nuclear Power Plant Main Steam Pipeline. U.S. Patent.

[6] Tian M., Cong T., Ma Z., Chen R., Tian W., Qiu S., Su G.H. (2017). A new unidentified leak detection method based on the EVR ventilation condensate. Prog. Nucl. Energy.

[7] Zheng Y., Hu D., Dai Y. (2016). Simulation of the airborne radioactive substance distribution and monitoring of coolant leakage in a typical Nuclear Reactor Containment. Ann. Nucl. Energy.

[8] Jang J., Jeong J.Y., Park J., Cho Y.-S., Pak K., Kim Y.K. (2022). Feasibility study of β-ray detection system for small leakage from reactor coolant system. Nucl. Eng. Technol..

[9] Broek D., Broek D. (1982). Fracture of structures. Elementary Engineering Fracture Mechanics.

[10] Kobayashi A.S., Ziv M., Hall L.R. (1965). Approximate stress intensity factor for an embedded elliptical crack near two parallel free surfaces. Int. J. Fract. Mech..

[11] Jiang Y., Xia H., Wang Z., Zhang J., Yin W. Research and Design of LBB System for Main Pipeline of Nuclear Power Plant. Proceedings of the 2021 28th International Conference on Nuclear Engineering.

[12] Zhang J., Chen R.H., Wang M.J., Tian W.X., Su G.H., Qiu S.Z. (2017). Prediction of LBB leakage for various conditions by genetic neural network and genetic algorithms. Nucl. Eng. Des..

[13] Wichman K., Lee S. (1990). Development of USNRC standard review plan 3.6.3 for leak-before-break applications to nuclear power plants. Int. J. Press. Vessels Pip..

[14] Waters L.S., McKinney G.W., Durkee J.W., Fensin M.L., Hendricks J.S., James M.R., Johns R.C., Pelowitz D.B. (2007). The MCNPX Monte Carlo Radiation Transport Code. AIP Conf. Proc..

[15] Sallaberry C.J., Kurth R., Kurth-Twombly E., Brust F.W. (2021). Probabilistic Leak-Before-Break Evaluations of Pressurized-Water Reactor Piping Systems Using the Extremely Low Probability of Rupture Code.

[16] Program 41.01.04: Pressurized Water Reactor Materials Reliability Program (MRP)|Product Abstract. https://www.epri.com/research/programs/061145/results/3002023872.

[17] Appendix A to Part 50—General Design Criteria for Nuclear Power Plants. https://www.nrc.gov/reading-rm/doc-collections/cfr/part050/part050-appa.html.

[18] Rondeau A., Lafargue E., Weilemann A., Cartier F. (2019). Study of the health of an operating industrial valve by means of the continuously acoustic emission measurement. Proc. Meet. Acoust..

[19] FLÜS: Leak Detection System for Components and Compartment Humidity Monitoring. https://www.framatome.com/solutions-portfolio/portfolio/product/A0821/fl-s-leak-detection-system-for-components-and-compartment-humidity-monitoring.

[20] Fernlund E. A Novel Pump-Controlled Asymmetric Cylinder with Electric Regeneration: Implementation and Evaluation of a Closed Hydraulic System on a Backhoe. Proceedings of the 17th Scandinavian International Conference on Fluid Power.

[21] Carlstedt A. (2021). Modelling of Electromechanical Motors for Turret and Barrel Control in Main Battle Tanks. https://www.diva-portal.org/smash/get/diva2:1595624/FULLTEXT01.pdf.

[22] Gayan S., Senanayake R., Inaltekin H., Evans J. (2021). Reliability Characterization for SIMO Communication Systems with Low-Resolution Phase Quantization Under Rayleigh Fading. IEEE Open J. Commun. Soc..

[23] Amree C., Chaijit S. Strain Hardening Analysis of SUS 304 Stainless Steel Cup for Multi Stage Deep Drawing Using Finite Element Simulation Comparison with Experiment Result. Proceedings of the 2018 2nd International Conference on Engineering Innovation (ICEI).

[24] Srisa-An C. Guideline of Collinearity—Avoidable Regression Models on Time-series Analysis. Proceedings of the 2021 2nd International Conference on Big Data Analytics and Practices (IBDAP).

[25] Chohan D.K., Dobhal D.C. A Comparison Based Study of Supervised Machine Learning Algorithms for Prediction of Heart Disease. Proceedings of the 2022 International Conference on Computational Intelligence and Sustainable Engineering Solutions (CISES).

[26] Alibrahim H., Ludwig S.A. Hyperparameter Optimization: Comparing Genetic Algorithm against Grid Search and Bayesian Optimization. Proceedings of the 2021 IEEE Congress on Evolutionary Computation (CEC).

